# Early Transcriptional Liver Signatures in Experimental Visceral Leishmaniasis

**DOI:** 10.3390/ijms22137161

**Published:** 2021-07-02

**Authors:** Génesis Palacios, Raquel Diaz-Solano, Basilio Valladares, Roberto Dorta-Guerra, Emma Carmelo

**Affiliations:** 1 Instituto Universitario de Enfermedades Tropicales y Salud Pública de Canarias (IUESTPC), Universidad de la Laguna (ULL), Avenida Astrofísico Francisco Sánchez s/n, 38200 La Laguna (Tenerife), Spain; gpalacio@ull.edu.es (G.P.); rdiazsol@ull.edu.es (R.D.-S.); bvallada@ull.es (B.V.); rodorta@ull.edu.es (R.D.-G.); 2 Departamento de Obstetricia y Ginecología, Pediatría, Medicina Preventiva y Salud Pública, Toxicología, Medicina Legal y Forense y Parasitología, Universidad de La Laguna, Avda. Astrofísico F. Sánchez s/n, 38200 La Laguna (Tenerife), Spain; 3 Red de Investigación Colaborativa en Enfermedades Tropicales (RICET); 4 Departamento de Matemáticas, Estadística e Investigación Operativa, Facultad de Ciencias, Universidad de La Laguna, 38200 La Laguna (Tenerife), Spain

**Keywords:** transcriptional profiling, high-throughput RT-qPCR, multivariate analysis, principal component analysis, *Leishmania infantum*, gene expression, gene signature, liver, mouse experimental model

## Abstract

Transcriptional analysis of complex biological scenarios has been used extensively, even though sometimes the results of such analysis may prove imprecise or difficult to interpret due to an overwhelming amount of information. In this study, a large-scale real-time qPCR experiment was coupled to multivariate statistical analysis in order to describe the main immunological events underlying the early *L. infantum* infection in livers of BALB/c mice. High-throughput qPCR was used to evaluate the expression of 223 genes related to immunological response and metabolism 1, 3, 5, and 10 days post infection. This integrative analysis showed strikingly different gene signatures at 1 and 10 days post infection, revealing the progression of infection in the experimental model based on the upregulation of particular immunological response patterns and mediators. The gene signature 1 day post infection was not only characterized by the upregulation of mediators involved in interferon signaling and cell chemotaxis, but also the upregulation of some inhibitory markers. In contrast, at 10 days post infection, the upregulation of many inflammatory and Th1 markers characterized a more defined gene signature with the upregulation of mediators in the IL-12 signaling pathway. Our results reveal a significant connection between the expression of innate immune response and metabolic and inhibitory markers in early *L. infantum* infection of the liver.

## 1. Introduction

Visceral leishmaniasis (VL) is one of the clinical forms of leishmaniasis, caused mainly by the intracellular protozoan *Leishmania donovani* or *Leishmania infantum*. Although there exist many experimental animal models for VL (e.g., rodents, dogs, monkeys, etc.), none of these models accurately reproduces what happens in humans (reviewed in [1]). The Syrian golden hamster is considered to be the best experimental model to study VL (reviewed in [1]), since it closely mimics the clinicopathologic features observed in naturally infected dogs and humans. However, the use of Syrian hamster models is limited due to the unavailability of some reagents [2,3]. The murine animal model (mainly the BALB/c and C57BL/6 strains) has been extensively used in the immunological study of this disease. BALB/c is a susceptible strain that develops a life-long chronic infection which is not fatal to the host [3]. Although experimental murine models of VL do not allow exact extrapolations with subclinical infection in humans, they have nonetheless been useful in identifying genes and predicting their functional roles in protective immune response (reviewed in [1]). For these reasons, we decided to use BALB/c as the experimental model in this study. When the infection is reproduced in an experimental model, an immune response occurs in the main affected organs, liver and spleen. In the liver, the formation of cellular infiltrates or inflammatory granulomas occurs around the infected macrophages. Kupffer cells (KC) are key in this process, since these are the main tissue macrophages that cover hepatic sinusoids and are the main target of infection by strains of *Leishmania* causing VL [4,5].

The study of transcriptional changes at the tissue level represents a significant strategy to evaluate host–pathogen interactions and to elucidate the mechanism of pathogenesis of experimental VL. Many studies have focused on the transcriptomic analysis of immunological response in the spleen in *Leishmania*-infected experimental hosts [6,7,8,9], where many mediators were found to play a role in M1/M2 and Th1/Th2 responses, and also markers of T reg, Th17, and Tfh cells are involved (reviewed in [10]). However, transcriptomic analysis regarding immunological response mechanisms in the liver (where infection develops shortly after inoculation) are scarce [9]. Additionally, most studies in infected livers have been carried out after the acute phase when parasitic load is plateauing and infection control is underway [9]. Interestingly, transcriptomic analysis of the immunological response to *L. donovani* infection using blood, spleen, and liver tissue of BALB/c mice has shown a remarkably common signature with the upregulation of interferon responsive genes in all tissues [9]. These sort of studies are useful for the identification of the pathways and immunological processes involved in infection, and have been used in the search for immunomodulatory strategies [6,11,12]. One example of this in experimental VL is type I interferon signaling. Manipulation of this pathway was found to be an encouraging strategy for improving disease outcomes in VL patients [13]. The in-depth analyses of the processes that drive early infection will provide a better insight into the immunological strategies that could control *L. infantum* infection.

The methodology used in this study is based on high-throughput real-time qPCR for the amplification of 223 genes related to both the immune response and lipid and carbohydrate metabolism. This is a highly sensitive and specific technology designed to perform large-scale gene expression analyses, allowing the correction of experimental variations by normalization. High-throughput real-time qPCR presents one important advantage over the most widely used methodologies in transcriptomics (RNA-seq, microarrays, etc.), namely that the results do not require validation by qPCR. Gene expression is also well-correlated with protein expression for numerous markers (such as IL12, IL10, IFN-γ, TLR2-4, TNF-α) in murine models [14]. In this large-scale analysis, three different parameters were integrated: parasite burden in liver tissue, the weight of the organ, and gene expression profile in the liver at different timepoints. This large dataset was streamlined using principal component analysis (PCA) in order to identify a particular gene signature for some timepoints based on the contribution of each gene’s expression. PCA analysis revealed strikingly different gene-expression patterns between timepoints, dependent on the molecular processes taking place during early infection. In our experiments, at one day post inoculation (p.i.), a number of genes involved in interferon signaling pathway and cell chemotaxis were upregulated. However, at ten days p.i., the gene signature was characterized by a focused response including upregulation of genes involved in the interleukin-12 signaling pathway. The expression of inhibitory and metabolism markers was also assessed in the context of early *L. infantum* infection.

## 2. Results 

### 2.1. Leishmania Infantum Infection Is Established in Liver 24 h Post Inoculation

**Figure 1:**
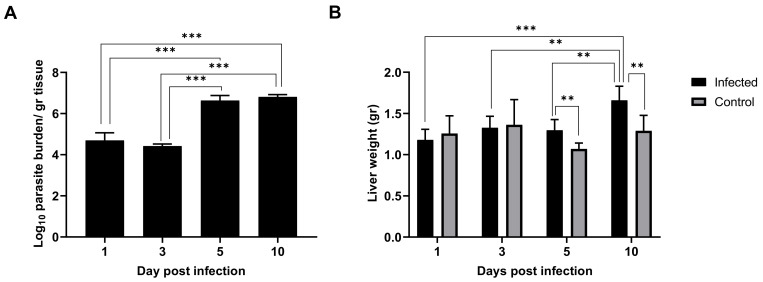
*Leishmania infantum* infection is established in liver 24 h post inoculation: (**A**) Evolution of parasite burden in liver. Mice were inoculated intravenously (i.v.) with 1 × 10^6^ promastigotes. Parasite load was determined 1-, 3-, 5- and 10-days post infection by limiting dilution assay and expressed as log_10_ of the average parasite load per gram of tissue. (**B**) Evolution of liver weight in infected (*n* = 24) vs. control mice (*n* = 24) over the course of infection. Error bars indicate Standard Error of Mean (SEM). One-way analysis of variance (ANOVA) was used to compare the differences in parasite burden between timepoints. Two-way ANOVA was used to compare the weight of liver between infected and control mice at each timepoint. Statistically significant differences are indicated (** *p* < 0.01, *** *p* < 0.001).

As early as 24 h post inoculation, high parasitic burden was detected in the liver tissue, indicating that the infection was already established in this organ (Figure 1A). This parasitic burden remained stable until three days p.i. At five days p.i., parasitic burden jumped two orders of magnitude and remained elevated subsequently. Hepatomegaly in the infected mice groups was not evident one and three days p.i., but small differences arose at five days p.i. and particularly at ten days p.i. (Figure 1B), which is in agreement with the leap in parasitic burden observed (Figure 1A). Given our results, it seems clear that although the liver was already colonized 24 h after *L. infantum* inoculation, clinical signs (such as patent inflammation) appeared a few days later in the infection in BALB/c mice.

### 2.2. Multivariate Analysis Identified Particular Gene Expression Profiles at 1- and 10-Days Post Infection

**Figure 2:**
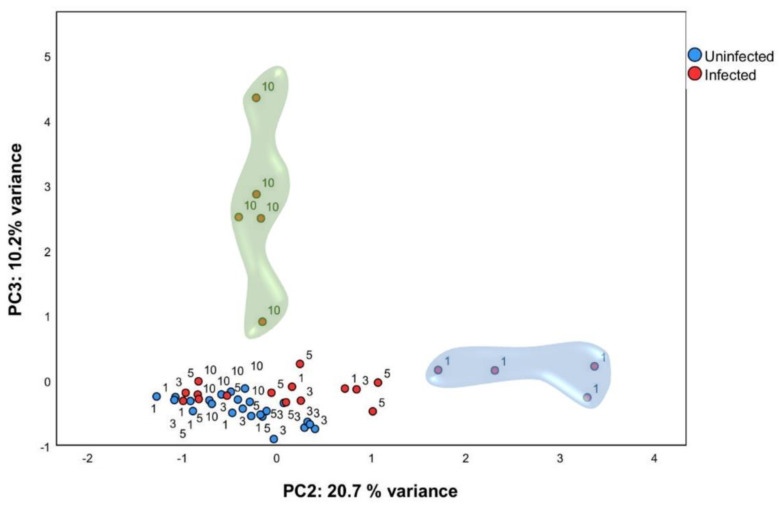
Multivariate analysis identified particular gene expression profiles at one and ten days post infection. qPCR was carried out on liver tissue from control and *L. infantum*-infected mice and at 1, 3, 5, and 10 days post infection. Principal component analysis (PCA) plot. Principal components scores are shown on the X and Y axis, respectively, with the proportion of total variance related to that principal component (PC) indicated. Each dot represents a mouse in the corresponding day, and the color indicates the condition (red, infected; blue, control). The clouds highlight the group of ten days p.i. mice by PC3 (green cloud) and the group of one day p.i. mice by PC2 (blue cloud).

In order to identify gene expression profiles in this large dataset, PCA analysis was performed using normalized relative quantity (NRQ) of all infected (*n* = 24) and control (*n* = 24) mice at all timepoints (Figure 2). Four components were extracted with 60.84 % (cumulative) of the total variance explained. Principal component 2 (PC2) and principal component 3 (PC3) were used for subsequent analysis (Table 1). PCA analysis showed that, one day post infection, mice infected with *L. infantum* formed a subset (Figure 2, indicated by a blue cloud). This was confirmed by two-way analysis of variance ANOVA (*p* < 0.01), having the score of PC2 as the response variable and time post infection (1, 3, 5, and 10 days post infection) and condition (either infected or control) as factors. Therefore, as early as 24 h post-inoculation, infected mice showed a clearly divergent gene-expression profile driven by the expression of genes correlated to PC2 as a response to infection by *L. infantum* (Table 1).

PCA analysis also showed that *L. infantum* infected mice at ten days p.i. form another subset (Figure 2, indicated by a green cloud). Using the score of PC3, we confirmed statistically significant differences between ten days p.i. mice and the control mice (*p* < 0.001), showing that infected mice also had a clearly divergent gene-expression profile at this timepoint with the expression of genes included in PC3 (Table 1). Additionally, unsupervised hierarchal clustering of the NRQ values was applied and, based on Euclidean distance and pairwise average linkage, was performed in order to visualize the relationships within this experimental dataset (Figure S1). Genes related to PC2 and PC3 are listed in Table 1 and were classified depending on whether they are involved in specific biological processes or pathways. The list of genes correlated with PC2 include genes related to the immunological response triggered by infection, such as M1/M2 polarization [8,15,16,17,18,19,20], but also genes involved in prostaglandin synthesis [21,22,23], lipid metabolism [24,25], MAPK signaling pathway [26], and regulatory T cell function [27] among others (Table 1). This indicates a very early activation (24 h after infection) of diverse signals. In contrast, genes correlated with PC3 were not as numerous, and most of them are involved in Th1 response, suggesting the gene signature is tipping over to a more defined response at 10 days post infection. The most striking findings will be described below.

### 2.3. Multiple Processes Are Activated in L. infantum Infection in Livers of BALB/c Mice at 1-Day Post Inoculation

**Figure 3:**
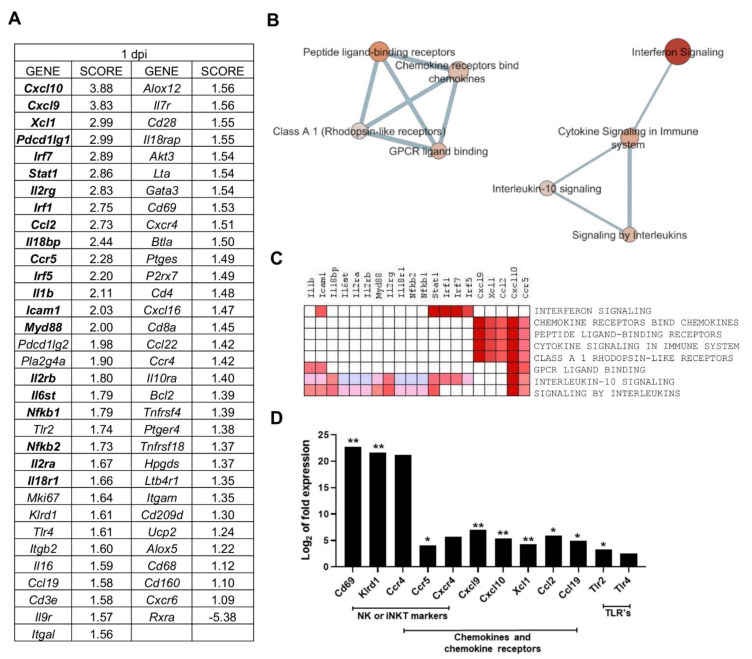
Multiple processes are activated in *L. infantum* infection in livers of BALB/c mice at 1-day post inoculation: (**A**) Ranked gene list generated in Gene Set Enrichment Analysis (GSEA) (using Principal Component 2-correlated genes to compare gene expression in 1 day p.i.-mice group vs. all other mice). Genes indicated in bold were involved in the core enrichment; (**B**) Enrichment Map with Principal Component 2 (PC2)-correlated genes. Parameters to filter these nodes/gene sets applied in Cytoscape: *p*-value < 0.05 and False Discovery Rate (FDR) < 0.25. Intensity of node color fill represents FDR value (more intense red color indicates lower FDR); node size corresponds to the Normalized Enrichment Score (NES) value. Thicker connection lines represent higher similarity coefficient between nodes; (**C**) Core enrichment of PC2-gene sets. The leading edge analysis was performed in GSEA with the gene sets enriched (with *p*-value < 0.05 and FDR < 0.25). The heat map shows the clustered genes in the leading edge subsets. Score values in the ranked gene list generated in GSEA are represented as colors, where the range of color (red, pink) shows the range of score values (high, moderate); (**D**) mRNA expression of genes at 1-day post infection *expressed* as Log_2_ of expression fold-change. Statistically significant differences between non- infected (control) and infected animals are indicated (* *p* < 0.05; ** *p* < 0.01).

Gene Set Enrichment Analysis (GSEA) was applied to PC2-correlated genes (65 genes), to identify the most relevant gene-expression signatures in one day p.i. mice (Table 2). GSEA software ranked genes according to their differential expression between the groups compared (one day p.i. mice group vs. all other mice) (Figure 3A). *Cxcl10* and *Cxcl9* had the highest score in the ranked list (applying tTest), revealing the strong correlation between these genes expression and the gene signature observed for one day p.i. mice group (Figure 3A). 

Enrichment analysis showed that eight gene sets were significantly enriched at FDR < 25% and nominal *p*-value < 5% (Table 2). The interferon signaling pathway was the most highly enriched pathway in one day p.i. mice, showing the highest NES value (normalized enrichment score, which was used to compare analysis results across gene sets [28]) and lowest FDR. This gene set is linked to *Cytokine signaling in the immune system* pathway, which in turn connects to signaling by interleukins and the interleukin-10 signaling pathway, according to the enrichment map (Figure 3B). The observed enrichment is related to the upregulation of key genes of those pathways (Figure 3C) and is characteristic of the one day p.i. mice group. 

The second annotation with highest NES value was the Chemokine receptors bind chemokines pathway (Table 2), forming a separate cluster connecting with several other annotations that involve processes of ligand-binding receptors (Figure 3B). The overrepresentation of the “Chemokine receptors bind chemokines” Reactome pathway is in agreement with the upregulation of chemokines and chemokine receptors such as *Cxcl10*, *Cxcl9*, *Xcl1*, *Ccl2*, and *Ccr5* (Figure 3D). These genes were the core enrichment of this pathway, as described by GSEA, and contribute most to the enrichment result of this gene set (Figure 3C). The upregulation of these genes is one of the earliest outcomes of *Leishmania* infection described both in vitro and in vivo [29,30,31,32,33]. This chemokine response at one day p.i. can be triggered by upstream signalling by toll-like receptors like *Tlr2* and *Tlr4* upregulated at this timepoint (Figure 3D). In addition, some markers involved in toll-like receptor signaling and intracellular signaling (*Il1b*, *Icam1*, *Il18bp*, *Il6st*, *Il2ra*, *Il2rb*, *Myd88*, *Il2rg*, *Il18r1*, *Nfkb1*, and *Nfkb2*) were found in the core enrichment of the overrepresented “signaling by interleukins” annotation (Figure 3C).

**Figure 4:**
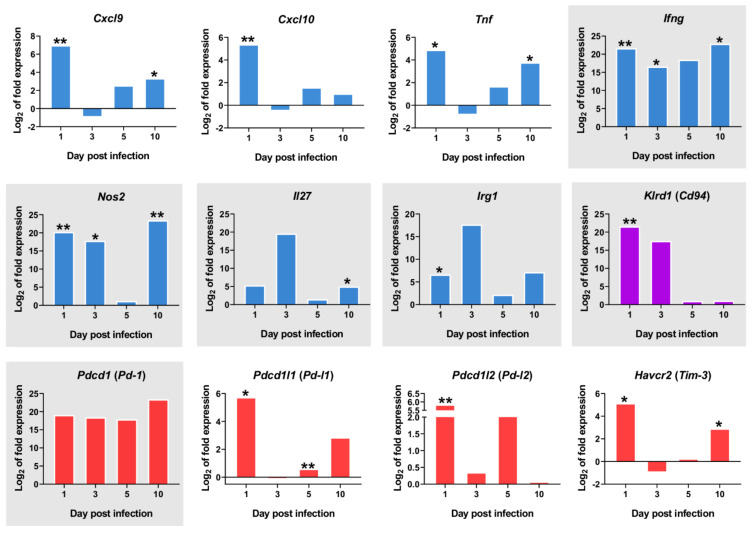
mRNA expression of important markers overtime. The y-axis represents log_2_ of expression fold-change for each indicated gene, that is the ratio between the average gene expression in the infected group and non-infected-control mice. The x-axis represents time after infection 1, 3, 5, and 10 days post infection. M1-associated transcripts (blue bars), inhibitory markers (red bars), *Klrd1* (*Cd94*) expression (purple bars). Graphics with gray background represent genes with Log_2_FC>1 in all timepoints. Statistically significant differences between non-infected (control) and infected animals are indicated (* *p* < 0.05; ** *p* < 0.01).

A number of other PC2-correlated genes show a clearly differential expression in the one day p.i.-mice group when compared to the other timepoints. Several of these correspond to genes expressed in natural killer (NK) cells or invariant natural killer T (iNKT) cells, such as *Cd69*, an iNKT cell marker, *Klrd1* (alias *Cd94*, an NK cell marker), *Ccr5*, *Ccr4* and *Cxcr4* (Figure 3D). Despite the observed upregulation of M1 -associated transcripts at 1 day p.i. (*Cxcl9, Cxcl10, Ifng, Tnf*) (Figure 4), there was also strong upregulation of inhibitory ligands such as *Pd-l1* (*Pdcd1lg1*) and *Pd-l2* (*Pdcd1lg2*), *Havcr2* (*Tim-3*), and *Pd-1* (*Pdcd1*) (Figure 4). 

The overrepresentation of *Interferon Signalling pathway* (FDR = 0.039) with the upregulation of *Stat1, Irf1, Irf7, Irf5, Icam1* (Figure 3C) and *Chemokine receptors bind chemokines* pathway (FDR = 0.199) with the upregulation of *Cxcl10, Cxcl9, Xcl1, Ccl2, Ccr5* (Figure 3C) constitute the gene-expression signature 24 h after *L. infantum* infection in the livers of BALB/C mice.

### 2.4. Downregulation of Transcriptional Factors Involved in Lipid Metabolism and Inflammation

**Figure 5:**
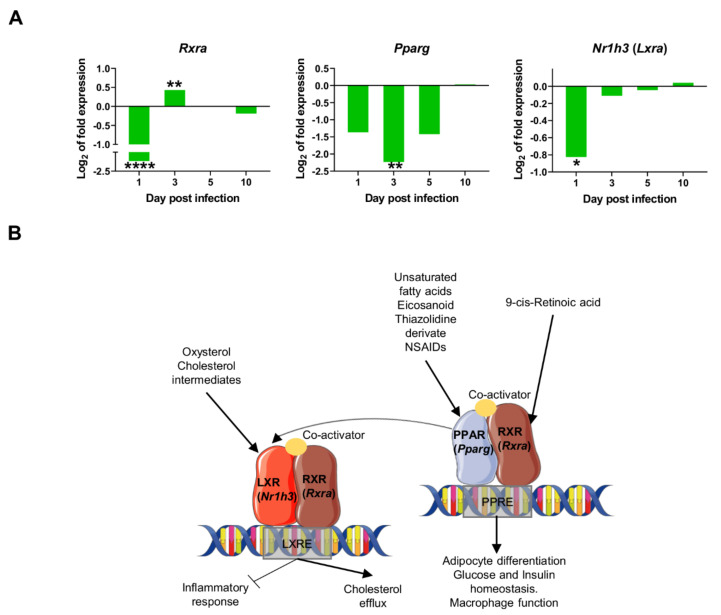
Downregulation of transcriptional factors involved in lipid metabolism and inflammation: (**A**) mRNA expression of genes at 1, 3, 5, and 10-days post infection, expressed as Log_2_ of expression fold-change. Statistically significant differences between non-infected (control) and infected animals are indicated (* *p* < 0.05; ** *p* < 0.01; **** *p* < 0.0001); (**B**) In the macrophage nucleus, Nuclear Receptors (NR’s) Peroxisome proliferator-activated receptors (PPARs) and Liver X receptors (LXRs) bind to specific response elements (PPAR response element (PPRE) or LXR response element (LXRE)) in target genes as heterodimers with retinoid X receptors (RXRs). Activation of these NRs results in transrepression of proinflammatory genes (*Nos2, Ptgs2*) but activation of cholesterol efflux, adipocyte differentiation, glucose, and insulin homeostasis, among others. This signaling is activated by several molecules like 9-cis-retinoic acid, unsaturated fatty acids, eicosanoids, oxysterol, and cholesterol intermediates. In our model, the downregulation of nuclear receptors (*Pparg, Rxra, Nr1h3*) was observed at some timepoints shortly after infection, consistent with the overexpression of proinflammatory markers observed as early as one day post infection [35,46,47].

In contrast to the positive correlation of most PC2 genes, *Rxra* was the only gene negatively correlated with PC2 (Table 1). Therefore at one day post infection (and only at this time of infection), the particular gene-expression signature observed in infected mice was dependent on the upregulation of PC2 genes, and downregulation of *Rxra. Rxra* codes for Retinoid X Receptor Alpha (RXR-α), a nuclear receptor that mediates the biological effects of retinoids by binding, either as homodimer or heterodimer, to specific sequences in the promoters of target genes (Figure 5B). RXR-α forms functional heterodimers with Peroxisome proliferator activated receptor (PPAR) proteins in a key pathway for lipid metabolism, adipocyte differentiation, and glucose and insulin homeostasis, among other functions (reviewed in [34]). RXR-α also forms heterodimers with LXRA (Liver X Receptor-Alpha coded by *Nr1h3* gene) to bind LXR response elements (LXREs), regulating the expression of genes involved in macrophage cholesterol and fatty acid metabolism [35]. Ligand activation of PPAR*γ* has also been shown to induce LXR target genes and to promote cholesterol efflux in the presence of LXR ligands. Similarly, PPAR*γ* and LRX ligands have been described to have anti-inflammatory effects, both in vitro and in vivo, through the inhibition of expression of genes involved in inflammation and innate immune response, such as iNOS, COX-2, TNF-*α*, IL-6, IL1-*β* among others [36,37,38,39,40]. PPAR*γ* activation downregulates proinflammatory cytokines in macrophages exposed to *L. infantum*-Lipophosphoglycan (LPG), a surface molecule only found in promastigotes [41]. In visceral and cutaneous leishmaniasis, several studies have demonstrated that the activation of macrophage PPAR*γ* promotes disease progression, and its inhibition delays lesion development [42,43,44] (reviewed in [45]). In liver granulomas, it has been shown that sustained RXR-α /PPAR signalling is important for parasite growth and survival, and that infected macrophages are less susceptible to being polarized towards an M1 phenotype [45] Similarly, pharmacological inhibition of RXR-α results in reduced parasite load in the spleen and liver [25]. 

Our data reveal the strong downregulation of several transcription factors at 1dpi: *Rxra, Pparg*, and *Nr1h3* (*Lxra*) (Figure 5A), thereby suggesting an early inhibition of lipid metabolism routes like cholesterol efflux and fatty acid metabolism. This downregulation is transient since expression of all three markers return to control levels by 10 days post infection. The downregulation of those nuclear receptors probably contributes to the transcriptional induction of pro-inflammatory genes in the early innate immune response in the infection by *L. infantum*, such as *Ifng, Nos2*, and *Tnf* (Figure 4).

### 2.5. L. infantum Infection Induces Th1 Responses through Interleukin-12 Signaling at 10 Days Post Infection

**Figure 6:**
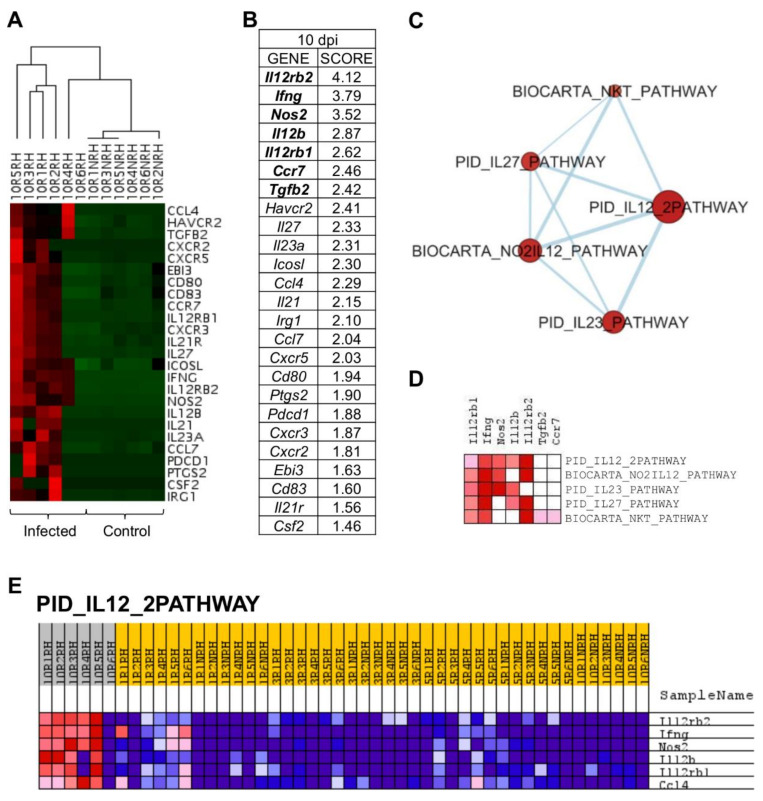
*L. infantum* infection induces Th1 responses through interleukin-12 signaling at 10 days post infection: (**A**) Unsupervised hierarchal clustering of 10 days p.i. and control mice including genes correlated with Principal Component 3 (PC3) was applied and based on Euclidean distance and pairwise average-linkage. Differential expression of selected genes in 10 days p.i.-mice (left side) and control mice (right side) is shown. Each mouse is identified with a respective code. Genes downregulated during infection are shown in green and upregulated genes are in red; (**B**) Ranked gene list generated in GSEA (using PC3-correlated genes to compare gene expression in 10 days p.i.-mice vs. all other mice). Genes indicated in bold were involved in the core enrichment; (**C**) Enrichment Map with PC3-correlated genes shows the relevance of IL-12 signaling pathway at 10 days p.i. Parameters to filter these nodes/gene sets applied in Cytoscape: *p*-value < 0.05 and FDR < 0.25. Intensity of node color fill represents FDR value (more intense red color indicates lower FDR); node size corresponds to the NES value. Thicker connection lines represent higher similarity coefficient between nodes; (**D**) Core enrichment of PC3-gene sets. The leading edge analysis was performed in GSEA with the gene sets enriched (with *p*-value < 0.05 and FDR < 0.25). The heatmap shows the clustered genes in the leading edge subsets. Score values in the ranked gene list generated in GSEA are represented as colors, where the range of color (red, pink) shows the range of score values (high, moderate); (**E**) PID_IL12_2PATHWAY heatmap. This gene set was upregulated in 10 days p.i.-mice (PC3 dataset). Upregulation of these genes in 10 days p.i.-mice (left side) and the rest of mice is shown. Each mouse is identified with a respective code. Genes are ordered by tTest ratio according to their differential expression. Normalized relative quantities (NRQ) are represented as colors, where the range of colors (red, pink, light blue, dark blue) shows the range of expression values (high, moderate, low, lowest).

PCA allowed the discrimination of infected mice ten days post inoculation (Figure 2). This differentiation owes to the upregulation of some genes at that timepoint (Figure 6A), that are the genes correlated with PC3 in the principal component analysis (Table 1).

Gene set enrichment analysis (GSEA) was applied to PC3-correlated genes (25 genes), revealing that *Il12rb2*, *Ifng*, *Nos2*, *Il12b*, *Il12rb1*, *Ccr7*, and *Tgfb2* had the highest score in GSEA ranked gene list (Figure 6B) and, therefore, that they are key to the particular gene signature of 10 days p.i.-mice compared to the rest of mice in the experiment. An enrichment map was constructed using PC3-correlated genes to visualize the main functional categories (Figure 6C). Under these conditions, only five gene sets were upregulated by PC3 genes (Table 3). IL12-mediated signaling events (PID_IL12_2PATHWAY) was the annotation with the highest NES value and lowest FDR. Remarkably, genes involved in “PID_IL12_2PATHWAY” were upregulated in 10 days p.i.-mice group but not in the rest of the mice (Figure 6E), indicating their key role at this time of infection. The core enrichment of this pathway includes *Il12rb2, Ifng, Nos2, Il12b*, and *Il12rb1* (Figure 6D). Other pathways upregulated in 10 days p.i.-mice group were related to IL23-mediated signaling events (PID_IL23_PATHWAY) with the upregulation of *Ifng, Il12rb1, Il12b, Nos2*, and *Il23a*. Additionally, IL27-mediated signaling pathway (PID_IL27_PATHWAY) was overrepresented, including *Il12rb2, Ifng, Il12b, Il12rb1, Il27*, and *Ebi3*. All these genes were correlated with PC3. 

Two pathways related to NK cells were overrepresented at this timepoint, due to the upregulation of characteristic markers: NO_2_-dependent IL-12 pathway in NK cells (BIOCARTA_NO2IL12_PATHWAY) (*Il12rb2*, *Ifng*, *Nos2, Il12rb1, Cxcr3*) and selective expression of chemokine receptors during T-cell polarization (BIOCARTA_NKT_PATHWAY) (*Il12rb2*, *Ifng*, *Il12rb1*, *Ccr7*, *Tgfb2, Cxcr3, Ccl4, Csf2*) (Figure 6A). The upregulation of markers involved in these pathways shows that macrophage activation is occurring at 10 days p.i. and the differentiation of CD4^+^ T cells is being promoted to the Th1 subset. Strikingly, the upregulation of inhibitory markers observed at one day p.i. is no longer evident at 10 days p.i. in livers of BALB/c infected mice (Figure 4). Among inhibitory markers, *Havcr2* (*Tim-3*) was the only one showing statistically significant upregulation at 10 days p.i. (*p* = 0.022).

In conclusion, the gene-expression signature predominant in livers of BALB/c mice 10 days after infection with *L. infantum* is characterized by the overrepresentation of *IL12-mediated signaling events* (FDR=0.003) with the strong upregulation of *Il12rb2*, *Ifng*, *Nos2*, *Il12rb1*, and *Il12b*.

## 3. Discussion

Numerous scientists have used transcriptional analysis as a tool to detect changes in complex biological scenarios, such as infection or candidate vaccine testing. Most of those analysis are either lacking in precision due to the limited number of genes analyzed, or, on the contrary, use RNA-seq methodologies that provide a plethora of different data, which sometimes difficult to relate to the topic of interest, and the results of which generally require confirmation by RT-qPCR [8,25,48,49]. In contrast, the development of high-throughput real-time qPCR platforms allows for large-scale gene-expression analysis of large collections of genes that can be used to identify global patterns [6]. In this paper, a multivariate statistical approach was applied to a large-scale gene expression experiment in order to streamline a large collection of RT-qPCR gene expression data and describe the immunological mechanisms underlying early *L. infantum* infection in livers of BALB/c mice. This approach simplifies the characterization of the most relevant changes at the transcriptome level and the connection with other data such as parasite burden and weight of organ, providing a more accurate insight on the changes over time during infection. The traditional analysis of Differentially Expressed Genes (DEGs) with thresholds for *p*-values and fold-changes (FC), can sometimes be arbitrary and lead to the loss of true DEGs (if too conservative) or their false inclusion (if too relaxed) [50]. Hence, we decided to use a multivariate statistical analysis (i.e., principal component analysis) for gene expression analysis instead of merely basing our analysis on DEGs. Enrichment analysis coupled with the results of principal component analysis was useful for the contextualization of genes into pathways [50].

Our study involved high-throughput real-time qPCR (more than 32000 qPCR reactions) in livers of *L. infantum* infected (and non-infected, as controls) BALB/c mice very early after inoculation (1, 3, 5, 10-days p.i.). Our analysis mapped global changes in gene expression profiles of 223 genes of innate and adaptive immune response, prostaglandin synthesis, C-type lectin receptors, lipid metabolism and MAPK signaling pathway. PCA analysis revealed infected mice at one and ten days post infection form two statistically significant independent groups, as a response to the expression of two separate sets of genes: those contributing to PC2 are overexpressed at one day p.i. (except *Rxra*) and those contributing to PC3 are overexpressed at ten days p.i. Therefore, we decided to base the analysis of the gene-expression signatures observed at those timepoints on the investigation of the molecular pathways and biological processes overrepresented in these gene sets through gene set enrichment analysis (GSEA). Ranked genes in GSEA allowed us to categorize genes according to their differential expression between the groups compared. In our case, this was either the one day p.i. or ten days p.i. mice group, compared to all other mice in our experiment. 

As early as 24 h after parasite inoculation in mice, results show the relevance of *Cxcl10* (IFN-γ Inducible Protein 10, IP-10) and *Cxcl9* (Monokine Induced by IFN-γ, MIG). These two chemokines were strongly upregulated at one day p.i., and they are critical in both innate and adaptive immune responses to *Leishmania* infection [51]. Interestingly, *Cxcl9* has been identified as a hub gene in the search of common response signatures in spleen, liver, and blood during murine *L. donovani* infection [10]. CXCL10 has been studied as a treatment in BALB/c mice infected by *L. infantum*, since there is evidence that CXCL10-treatment reduces IL-10^+^ Treg cell populations (CD4^+^CD25^+^Foxp3^+^ and Tr1) and induces morphological changes in the spleen [52]. This chemokine also induces a reduction in parasite burden in the spleen and a decrease in IL-10 and TGF-β production in *L. infantum*-infected BALB/c mice [53]. 

At this timepoint, the upregulation of *Cxcl10* and *Cxcl9* are chemotactic signals to NK cells to generate IFN-γ, in agreement with the high enrichment of the *Interferon signaling* pathway. It has been demonstrated that NK and NKT cells are sources of hepatic IFN-γ and this in turn, is responsible for the accumulation of CXCL10 mRNA at one day p.i. of hepatic *L. donovani* infection in C57BL/6 mice [29]. Once parasites are inoculated in the mouse and Kupffer macrophages uptake the parasites, one important cell population that participates at this early stage are natural killer T (NKT) cells [9,54] and, particularly, iNKT cells, a major lymphocyte subtype in the liver [54]. The strong upregulation of *Cd69* at one day p.i., an iNKT cell marker, suggests a key role for this cell type at this timepoint. Other genes correlated with PC2, such as *Klrd1 (Cd94)*, *Cxcr4, Ccr4*, and *Ccr5* are also expressed in NK cells [55]. It was demonstrated that IFNγ produced by NKT-cell-deficient mice is reduced by 50% compared to naïve [29]. The fact that NK cells are an important cell source of IFNγ in the innate immune response in the liver against *Leishmania sp*. infection is consistent with our results. Interestingly, Ashwin et al. demonstrated the upregulation of *Klrd1 (Cd94)* in advanced liver infections, but the downregulation in the spleen and blood in *L. donovani* infection (36 and 42 days p.i.) [9]. 

Besides *Cxcl10* and *Cxcl9*, other chemokines were found in the core enrichment of the *Chemokine receptors bind chemokines* pathway at one day p.i., such as *Ccr5* and *Ccl2* (chemoattractant of macrophages, monocytes, NK cells, and other CCR2-expressing leukocytes). CCL2 has been shown to play an important role in early immunity against cutaneous leishmaniasis [51,56]. These markers are probably secreted by the nascent granulomas (a nucleus of parasitized Kupffer cells surrounded by other immune cells) which start assembling once *Leishmania* parasites are transferred to the liver after inoculation [57]. Those structures are crucial to activate the parasite-killing abilities of Kupffer cells [57], and secrete chemokines and cytokines which recruit immune cells including monocytes, neutrophils, and invariant natural killer T (iNKT) cells [29,33]. The inflammatory chemokine response in Kupffer cells is driven by upstream signalling by toll-like receptors [58,59]. In our experiments, *Tlr2* and *Tlr4* were upregulated and correlated with PC2. *Tlr2* has been reported as potential therapeutic target in visceral leishmaniasis [60]. In livers of susceptible but self-curing C57BL/6 mice, *L. donovani* infection enhanced toll-like receptor 4 (TLR4) and TLR2 gene expression. In TLR2(−/−) mice, control of liver infection, parasite killing, and granuloma assembly has been accelerated and chemotherapy’s efficacy enhanced [60].

All this information shows that at one day p.i., the differential pattern of gene expression in infected mice is related to interferon signaling triggered by chemokine response. This is the first step in the activation of innate immunity by inducing production of proinflammatory cytokines to culminate in the release of reactive oxygen species (ROS) and NO produced by macrophages [61,62].

At ten days post infection, parasite load increased significantly (more than two orders of magnitude) compared to one and three days post infection. The statistically significant difference in the parasite burden between one and ten days post inoculation, in addition to the hepatomegaly observed in the ten days p.i. mice group, supports the divergent gene signature that differentiate these two groups of mice. This is consistent with the full maturation of granuloma from two to four weeks after infection [57]. Despite the fact that infection was clearly established as early as one day p.i. in the liver, hepatomegaly was only evident a few days later, reflecting the time needed for the clinical signs to appear.

The gene signature at 10 days p.i. is characterized by interleukin-12 stimulation for the production of IFN-γ, due to the upregulation of *I12b*, *Il12rb1*, and *Il12rb2* [63]. One of the main cytokines involved in the production of IFNG is IL12, which induces development of Th1 cells [64]. The upregulation of NO_2_-dependent IL12 Pathway in NK cells (BIOCARTA_NO2IL12_PATHWAY) also supports this, since secretion of IL12 by macrophages is essential for NK cells activation and the production of IFN-γ, that indeed stimulate the differentiation of Th1 cells. Similarly, the overrepresentation of BIOCARTA_NKT_PATHWAY, with the upregulation of *Cxcr3* (Th1-specific chemokine receptor (reviewed in [65]), suggests that there is active signaling of Th1 response cells at the site of the infection. These signals for leukocyte recruitment are key to trigger granuloma formation in the liver in the first weeks after infection. The upregulation of TNF also supports this, since this marker plays a crucial role in coordinating the assembly and maturation of granulomas (reviewed in [10,66]). These results show good agreement with the comparative analysis of the response to *L. donovani* infection at transcriptome level using liver tissue, spleen, and blood of BALB/c mice [9], where general upregulation of interferon responsive genes was described, as well as a significant upregulation of TNF, characteristic of liver infection. In our study, we also found *IL27-mediated signaling events* (PID_IL27_PATHWAY) overrepresented at 10 days p.i., probably due to the upregulation of *Il27* (p28) and *Ebi3*, both subunits of IL27. IL27 synergizes with IL12 the production of IFN-γ by CD4, CD8 T cells and NKT cells [67,68]. 

Altogether, the information derived from the expression profile of genes correlated with PC3 suggests that the gene signature at 10 days p.i. is clearly defined towards the activation of Th1 responses through interleukin-12 signaling.

The pro-inflammatory response activation is also supported by the downregulation of transcription factors such as *Pparg*, *Nr1h3* (*Lxr*), and *Rxra* early after infection. This downregulation is limited to the first days post infection, suggesting a transient infection-induced effect. The inhibition of these nuclear receptors is consistent with the upregulation of inflammatory markers. It has been demonstrated that the pharmacological manipulation of RXRα activity perturbs the transcriptomic network of infected KCs, leading to enhanced leishmanicidal activity [25]. Similarly, there is evidence that LXR-deficient mice are more resistant to *L. chagasi/infantum* infection compared to wild-type mice [69]. Our results confirm the relevance of *Rxra* and its counterparts *Nr1h3* (*Lxra*) and *Pparg* as early as one day post infection in livers of BALB/c mice, probably via the limitation of the pro-inflammatory response right after infection. These findings contribute to the mounting evidence that immune response and lipid metabolism are tightly linked in leishmaniasis and advocates for the use of metabolic regulators (such as retinoic acid) to counterbalance immunosuppression in leishmaniasis [70].

In addition to the upregulation of pro-inflammatory signals, several inhibitory markers, such as *Pd-l1* (*Pdcd1lg1*), and *Pd-l2* (*Pdcd1lg2*) were identified as key molecules, particularly at one day p.i. PD-L1 is expressed by a large range of cell types, including tumor cells, macrophages, monocyte-derived myeloid dendritic cells (DCs), epithelial cells, T cells, and B cells. In contrast, PD-L2 is induced by cytokines only on macrophages and DCs [71]. The receptor of those genes, PD-1, is induced by antigen stimulation [72] on peripheral CD4^+^ and CD8^+^ T cells, B cells, NK-T cells, and monocytes upon activation [73]. The differential expression of PD-1 ligands observed between infected and control mice is statistically significant early after infection and is probably explained by the activation of the early innate immune response. However, although upregulated, *Pd-1* differential expression is not statistically significant in our model, most likely because NK-T cells were probably the main cell type participating at one day p.i., according to our results, and these cells express low levels of PD-1 [74]. Besides *Pd-1* and its ligands, *Havcr2* (*Tim3*) has been considered as a checkpoint inhibitor [75] and is involved in T cell suppression in chronic viral infections [76] (reviewed in [77,78]). TIM-3 is expressed by T cells and NK cells, but also in myeloid cells such as DCs and monocytes. In our study, the statistically significant upregulation of *Havcr2* (*Tim-3*) at one and ten days p.i. is probably related with the evidence of NK cells relevance in the early infection. TIM-3 has been reported to be an exhaustion marker on NK cells; during treatment (ex vivo) with an anti-TIM-3 antibody, it reversed NK cell exhaustion in advanced melanoma [79]. The role of TIM-3 in *Leishmania* pathogenesis remains to be explored.

At one day post infection, the upregulation of those inhibitory genes (*Pd-l1, Pd-l2*, and *Tim3*) could be a response to counterbalance the upregulation of inflammatory markers, and therefore restrain the response to IFN-γ at this timepoint. The interactions PD-1/PD-L1 or PD-1/PD-L2 are well known to inhibit T cell effector functions including IFN-γ production and proliferation. In *L. donovani* infection (VL), the blockade of PD-1 [80] and/or PD-L2 [7] contribute to the reduction of parasitic load. In murine cutaneous leishmaniasis (CL), particularly in *L. amazonensis* infection, the blockade of PD-1 and PD-L1 reduced parasite load [81], and also increased IFN-γ-producing CD4^+^ and CD8^+^ T cells. The blockade of PD-1 pathway has been recognized an attractive strategy to improve prophylactic vaccination [75]. Few studies have focused on the PD-1 pathway during the early stages of T cell responses. Thus, we suggest the study of the contribution of NK cells to immunotherapy mediated by PD-1/PD-L1 blockade in leishmaniasis, as has been assessed in cancer [82]. Additionally, the role of the PD-1 pathway on CD4^+^ T cell differentiation should be better elucidated in leishmaniasis, because most of the studies on PD-1 pathway in leishmaniasis, have been focused on studying PD-1 blockade on CD8^+^ T cell responses [75].

The modulation of immune response in leishmaniasis has been explored as a strategy to enhance the host’s response during parasitic infection. Immunotherapeutic approaches include the use of cytokines or monoclonal antibodies, the combination of chemotherapy and immunotherapy, and the use of TLR agonists or small molecules. These strategies have been assayed in human and animal models with various outcomes (reviewed in [83,84]). In this paper we have described the most relevant gene-expression signatures observed during the very early stages of *L. infantum* infection in livers of BALB/c mice, including the differential gene-expression of some markers that may be used as pharmacological targets in order to stimulate innate immunity in infected animals. Among these, nuclear receptors *Rxra*, *Pparg*, and *Nr1h3* (*Lxra*) have been explored as a way to influence the outcome of different infectious diseases (reviewed in [85]). Nevertheless, given the multiple roles of these nuclear receptors in macrophage lipid metabolism, cholesterol efflux, inflammatory responses, and apoptosis, the use of any potential inhibitor must be carefully evaluated in order to avoid damaging essential cellular processes.

## 4. Materials and Methods

### 4.1. Biological Samples

Forty-eight female wild-type BALB/c mice were obtained from the breeding facilities of the Charles River Laboratories, (France) and were maintained under specific pathogen-free conditions at ULL. Half of these animals (*n* = 24) were randomly assigned to control group and the other half (*n* = 24) were infected by inoculation in the coccygeal vein of 10^6^ promastigotes of *L. infantum* (JPC strain, MCAN/ES/98/LLM-724) in stationary growth phase on day 0 of the experiment. *L. infantum* was maintained in vivo by serial murine passages. Prior to infection, amplification of amastigote-derived promastigotes, with less than three passages in vitro, was carried out by culture in RPMI medium (Gibco BRL, Grand Island, NY, USA), supplemented with 20% inactivated fetal calf serum (SBFI), 100 μg/mL streptomycin (Sigma-Aldrich, St. Louis, MO, USA) and 100 U/mL of penicillin (Biochrom AG, Berlin, Germany) at 26 °C until reaching stationary phase. 

At one, three, five, and ten days post-infection (days p.i.), six infected mice and six control mice (*n* = 12 each timepoint) were euthanized by cervical dislocation to extract the liver and proceed to the determination of liver weight and parasitic load [86]. Samples were immediately stored in RNA later at −80 °C (Sigma-Aldrich, St. Louis, MO, USA) for nucleic acid preservation and further mRNA extraction. 

### 4.2. RNA Isolation and Quantification 

Preserved liver tissue (7–10 mg) was homogenized with TRI Reagent (Sigma-Aldrich) in Lysing Matrix D columns (MP Biomedicals, Solon, USA) and FastPrep® System (ProScientific, Cedex, France). mRNA enrichment was performed using RNeasy Mini Kit (Qiagen, Hilden, Germany), following manufacturer’s instructions. *DeNovix DS-11 Spectrophotometer* (*DeNovix*) was used to quantify and evaluate RNA purity. Only samples with OD_260/280_ ratios between 2–2.2 and ratio OD_260/230_ between 1.8–2.2 were included in this study. The integrity of the RNA was determined using 2100 Bioanalyzer (Agilent Technologies, Santa Clara, USA) and RNA 6000 Nano Kit chips (Agilent Technologies, Santa Clara, USARNA integrity number (RIN) was >7 for all RNA samples included in this study. Additionally, agarose gel electrophoresis was performed to all RNA samples for further verification of integrity and quality.

### 4.3. Retrotranscription and High-Throughput Real-Time Quantitative PCR (RT-qPCR)

A High-Capacity cDNA Reverse Transcription kit (Thermo Fisher Scientific) was used to perform the retrotranscription using a Veriti® 96-Well Fast Thermal Cycler thermocycler (Thermo Fisher Scientific). Real Time qPCR was performed using OpenArray® plates with TaqMan probes (Thermo Fisher Scientific) for the amplification of 223 genes that were selected and related to innate and adaptive immune response, prostaglandin synthesis, lipid metabolism, C-type lectin receptors and MAPK signaling pathway. All primers and probes were commercially designed by Thermo Fisher Scientific (Table S1). Each plate allowed performing 3072 amplification reactions. The plate was organized in 48 subarrays, each with 64 nanopockets. PCR mixture was prepared following the manufacturer’s instructions and plates were loaded using the Accufill^TM^ System (Thermo Fisher Scientific). Each amplification reaction was performed in a volume of 33 nl. The thermal cycle and fluorescence detection were performed with the QuantStudio^TM^ 12K Flex Real-Time PCR System (Thermo Fisher Scientific) following manufacturer’s recommendations. Each subarray contained the primers together with a probe labeled with FAM at its 5’ end. Each sample was amplified in triplicate. *Cq* values produced by this platform are already corrected for the efficiency of the amplification [6,87] and candidate reference genes.

### 4.4. Data Pre-Processing and Processing

Data were exported from QuantStudio^TM^ 12K Flex Real-Time PCR System software (Thermo Fisher Scientific, v 1.3) to MS Excel. The arithmetic average quantitative cycle (Cq) was used for data analysis. *GenEX (MultiD*) software was used for data preprocessing and normalization. Reference genes selection for this dataset [87] was performed in *GenEx* using two different methods, geNorm (setting M-value lower than 0.5) and NormFinder, revealing two genes that showed the most stable expression: *Lta4h*, *P38mapk*. Normalization with these genes was performed to obtain normalized relative quantities (NRQ) values.

### 4.5. Statistical Analysis

Mean and its standard error (SEM) was calculated for each parameter measured. For all the analyses, outliers were assessed by inspection of boxplots and studentized residuals, data were checked for normal distribution by the Kolmogorov-Smirnoff test or Shapiro-Wilk test, as appropriate, and homogeneity of the variance by means of the Levene test.

The differences in parasite burden between time points were determined by a one-way Analysis of Variance (ANOVA) and a two-way ANOVA was used to compare the weight of the livers between infected and control mice at each time point. Tukey’s test and pairwise comparisons with Bonferroni adjustment as post hoc comparison techniques were carried out to compare the means from different groups.

NRQ values were used to perform a principal component analysis (PCA), an unsupervised multivariate method, which provides a first approximation to the statistical analysis of expression profiling data with the aim of generating several hypotheses of interest. In the PCA, the correlation between variables was assessed by inspection of the correlation matrix, the overall Kaiser-Meyer-Olkin (KMO) measure, and the Bartlett’s test of sphericity, indicating that the data were likely factorizable. 

Unsupervised hierarchal clustering of the autoescaled NRQ values was applied and based on Euclidean distance and pairwise average-linkage was performed in Cytoscape 3.8.0 (http://www.cytoscape.org/, accessed on 17 February 2021) [88] using the plugin clusterMaker [89].

The next step was a confirmatory study to test the hypothesis. Differences in Principal Component 2 (PC2) and Principal Component 3 (PC3) scores were determined by a two-way ANOVA to study the combined effects of both factors, time post infection (1, 3, 5, and 10 days post infection) and condition (infected or control), as well as their interactions, followed by Tukey´s post-hoc tests and pairwise comparisons with Bonferroni adjustment, as appropriate. 

Descriptive analysis, the evaluation of assumptions, ANOVAs, and principal component analysis were carried out using IBM^®^ SPSS^®^ version 26 (IBM Corporation, Armonk, NY, USA) statistical software.

NRQ values were also used for individual gene representation. The fold change of gene expression was calculated as the ratio between the average gene expression in the infected group and non-infected-control mice and expressed as Log_2_. Statistical differences in NRQ values were analyzed by two tail unpaired t-test or two tail Mann–Whitney U test, as appropriate, using GraphPad Prism version 9.1.1, GraphPad Software, San Diego, CA, USA (www.graphpad.com, accessed on 14 May 2021.

### 4.6. Enrichment Analysis

To analyze the potential biological processes and pathways regarding to PC2 and PC3-correlated genes, we used gene set enrichment analysis (GSEA) software (version 4.0.3, San Diego, CA, USA). This is a computational method that determines whether an a priori defined set of genes shows statistically significant differences between two biological states (e.g., phenotypes) [28]. Metric for ranking genes was tTest ratio. One-hundred-thousand gene permutations were used to generate a null distribution for ES (Enrichment score), then each pathway will attain a normalization enrichment score (NES). We performed the enrichment analysis using gene sets from Reactome Pathway Database for PC2-correlated genes and Canonical Pathways gene sets in *C2: curated gene sets collection* and *Hallmark gene sets in Molecular Signatures Database (MSigDB)* for PC3-correlated genes [90]. 

With enrichment results, the Cytoscape *3.8.2*. software [88] and the plugins that are part of the EnrichmentMap Pipeline Collection [91] were used to create enrichment maps in order to integrate the results from GSEA. The enrichment map was generated using only the gene-sets passing the thresholds: nominal *p*-value < 0.05, *q*-value < 0.25. The overlap coefficient was set to 0.35. 

## Figures and Tables

**Table 1:** Highest factor loadings of PC2 and PC3 from the Principal Component Analysis (PC2/PC3-correlated genes).

**PC2 Genes**					
**Gene**	**Factor Loading**	**Gene**	**Factor Loading**	**Gene**	**Factor Loading**
*Il18r1*	0.742	*Cd8a*	0.683	***Tlr2***	0.594
*Pdcd1lg2 (Pd-l2)* ^e^	0.738	*Cd4*	0.682	*Cd160*	0.592
*P2rx7 (P2x7)* ^a^	0.733	***Ptges*** ^a^	0.679	***Irf1***	0.591
*Bcl2*	0.723	***Il2rg***	0.678	***Myd88***	0.585
*Gata3*	0.72	*Ptger4* ^a^	0.677	*Pla2g4a* ^b^	0.582
*Il16*	0.719	*Tnfrsf18 (Gitr)* ^f^	0.668	***Stat1***	0.58
*Cd3e*	0.718	*Il2rb*	0.653	*Ccl2*	0.58
*Il2ra (Cd25)* ^f^	0.717	*Tnfrsf4 (Ox40)* ^f^	0.652	*Il18bp*	0.577
***Lta***	0.713	*Klrd1 (Cd94)* ^c^	0.639	***Irf5***	0.573
*Il10ra*	0.713	*Cd209d* ^c^	0.634	***Tlr4***	0.572
*Cd69*	0.712	*Itgam*	0.633	*Il6st*	0.572
***Akt3*** ^d^	0.707	***Icam1***	0.632	*Ltb4r1 (Blt1)* ^a^	0.571
***Cd28***	0.706	*Alox12* ^a^	0.632	***Il1b***	0.569
*Il7r (Cd127)*	0.704	*Xcl1*	0.622	***Nfkb1***	0.567
*Hpgds (Ptgds2)* ^a^	0.704	*Ucp2*	0.621	*Itgal*	0.567
*Mki67 (Ki67)*	0.702	*Pdcd1lg1 (Pd-l1)* ^e^	0.619	***Cxcl9***	0.567
*Ccr4*	0.701	***Cxcl10***	0.614	***Nfkb2***	0.566
*Il9r*	0.697	*Ccl22*	0.614	*Cxcl16*	0.562
***Cxcr4***	0.693	*Ccr5*	0.607	*Cxcr6*	0.552
*Ccl19*	0.689	***Irf7***	0.605	*Alox5* ^a^	0.544
*Btla*	0.685	*Itgb2*	0.603	*Rxra* ^b^	−0.569
*Il18rap*	0.684	*Cd68*	0.6		
**PC3 Genes**					
**Gene**	**Factor Loading**	**Gene**	**Factor Loading**	**Gene**	**Factor Loading**
*Il12rb1*	0.943	*Cxcr2*	0.825	*Cd83*	0.63
*Il12rb2*	0.941	***Il27***	0.803	***Csf2***	0.619
***Nos2***	0.928	***Cd80***	0.782	*Ccl4*	0.614
***Ccr7***	0.914	***Irg1 (acod1)***	0.768	*Havcr2 (Tim-3)* ^e,f^	0.591
***Il12b***	0.884	*Tgfb2*	0.693	*Icosl*	0.574
***Ifng***	0.867	*Cxcr3*	0.663	***Ebi3 (Il27b)***	0.562
*Cxcr5*	0.864	***Ccl7***	0.657	*Il21r*	0.558
***Il23a***	0.842	*Pdcd1 (Pd-1)* ^e,f^	0.643		
*Il21*	0.839	***Ptgs2*** ^a^	0.64		

M1-associated transcripts are shown in bold; M2-associated transcripts are in italics; ^a^ Prostaglandin synthesis; ^b^ Lipid Metabolism; ^c^ C-type lectin receptors (CRLs); ^d^ MAPK signaling pathway; ^e^ Inhibitory markers; ^f^ Regulatory T cell function.

**Table 2:** Gene sets upregulated in one day post infection-mice.

Nº	GENE SET NAME	RATIO	NES	NOM *p*-Value	FDR q-Value
1	INTERFERON SIGNALING (REACTOME R-HSA-913531,2)	5/149	2.050	0.000	0.039
2	CHEMOKINE RECEPTORS BIND CHEMOKINES (REACTOME DATABASE ID RELEASE 75 380108)	10/49	1.782	0.013	0.199
3	PEPTIDE LIGAND-BINDING RECEPTORS (REACTOME R-HSA-375276,5)	10/182	1.781	0.013	0.133
4	CYTOKINE SIGNALING IN IMMUNE SYSTEM (REACTOME DATABASE ID RELEASE 75 1280215)	32/608	1.708	0.024	0.170
5	CLASS A 1 RHODOPSIN-LIKE RECEPTORS (REACTOME R-HSA-373076,8)	12/307	1.629	0.036	0.227
6	GPCR LIGAND BINDING (REACTOME R-HSA-500792,3)	12/420	1.629	0.035	0.190
7	INTERLEUKIN-10 SIGNALING (REACTOME R-HSA-6783783,3)	6/42	1.584	0.040	0.212
8	SIGNALING BY INTERLEUKINS (REACTOME DATABASE ID RELEASE 75 449147)	25/417	1.580	0.047	0.189

Results of the enrichment analysis performed in GSEA software, filtered by *p*-value < 0.05 and FDR < 0.25.

**Table 3:** Gene sets upregulated in 10 days post infection-mice.

Nº	GENE SET NAME	RATIO	NES	NOM *p*-Value	FDR q-Value
1	PID_IL12_2PATHWAY (MSIGDB_C2 PID_IL12_2PATHWAY)	6/59	2.188	0.000	0.003
2	BIOCARTA_NO2IL12_PATHWAY (MSIGDB_C2 BIOCARTA_NO2IL12_PATHWAY)	5/15	1.965	0.003	0.013
3	PID_IL23_PATHWAY (MSIGDB_C2 PID_IL23_PATHWAY)	5/36	1.879	0.006	0.016
4	PID_IL27_PATHWAY (MSIGDB_C2 PID_IL27_PATHWAY)	6/25	1.788	0.012	0.024
5	BIOCARTA_NKT_PATHWAY (MSIGDB_C2 BIOCARTA_NKT_PATHWAY)	8/25	1.642	0.035	0.052

Results of the enrichment analysis performed in GSEA software, filtered by *p*-value < 0.05 and FDR < 0.25.

## Data Availability

Data used in this study have been deposited in GEO under accession code GSE159195.

## Supplementary Materials

The following are available online at https://www.mdpi.com/article/10.3390/ijms22137161/s1, Figure S1, Unsupervised hierarchal clustering of gene expression data; Table S1, List of TaqMan assays used for RT-qPCR analysis using Quant Studio^TM^ 12K Flex Real-Time PCR System.
