# Supplementary material for: Early Transcriptional Liver Signatures in Experimental Visceral Leishmaniasis
Source: Int J Mol Sci. 2021 Jul 2;22(13):7161. doi: 10.3390/ijms22137161 (PMC8267970; doi:10.3390/ijms22137161)
Supplement: Supplementary file 1 [file ijms-22-07161-s001.zip › ijms-1258368-supplementary.pdf]

Table S1. List of TaqMan assays used for RT-qPCR analysis using Quant Studio ™ 12K Flex Real-Time PCR System.

PANEL 1.

N°	Assay ID	GEN	NOMBRE DEL GEN	GRUPO
1	Mm00437762_m1	B2m	beta-2 microglobulin	Endogenous gene expression
2	Mm00446968_m1	Hprt	hypoxanthine guanine phosphoribosyl transferase	
3	Mm00435617_m1	Pgk1	phosphoglycerate kinase 1	
4	Mm01277042_m1	Tbp	TATA box binding protein	
5	Mm01201237_m1	Ubc	ubiquitin C	
6	Mm01722325_m1	Ywhaz	tyrosine 3-monooxygenase/tryptophan 5-monooxygenase activation protein, zeta polypeptide	
7	Mm00599890_m1	Ifngr1	interferon gamma receptor 1	Cytokines and cytokine receptors
8	Mm00492626_m1	Ifngr2	interferon gamma receptor 2	
9	Mm01168134_m1	Ifng	interferon gamma	
10	Mm01178820_m1	Tgfb1	transforming growth factor, beta 1	
11	Mm00436955_m1	Tgfb2	transforming growth factor, beta 2	
12	Mm00436964_m1	Tgfbr1	transforming growth factor, beta receptor I	
13	Mm00436977_m1	Tgfbr2	transforming growth factor, beta receptor II	
14	Mm00443258_m1	Tnf	tumor necrosis factor	
15	Mm00441875_m1	Tnfrsf1a	tumor necrosis factor receptor superfamily, member 1a	
16	Mm00441889_m1	Tnfrsf1b	tumor necrosis factor receptor superfamily, member 1b	
17	Mm01288580_m1	Irf1	interferon regulatory factor 1	
18	Mm00496 7_m1	Irf5	interferon regulatory factor 5	
19	Mm00516788_m1	Irf7	interferon regulatory factor 7	
20	Mm01224532_m1	Irg1	immunoresponsive gene 1	

21	Mm00518984_m1	Il23a	interleukin 23, alpha subunit p19	Interleukines and interleukin receptors
22	Mm00434237_m1	Il1r1	interleukin 1 receptor, type I	
23	Mm00439614_m1	Il10	interleukin 10	
24	Mm00434157_m1	Il10rb	interleukin 10 receptor, beta	
25	Mm00434169_m1	Il12a	interleukin 12a	
26	Mm00434174_m1	Il12b	interleukin 12b	
27	Mm00434189_m1	Il12rb1	interleukin 12 receptor, beta 1	
28	Mm00434200_m1	Il12rb2	interleukin 12 receptor, beta 2	
29	Mm00434204_m1	Il13	interleukin 13	
30	Mm00439618_m1	Il17a	interleukin 17A	
31	Mm00521423_m1	Il17f	interleukin 17F	
32	Mm00434214_m1	Il17ra	interleukin 17 receptor A	
33	Mm00434226_m1	Il18	interleukin 18	
34	Mm00456733_m1	Il18bp	interleukin 18 binding protein	
35	Mm00439620_m1	Il1a	interleukin 1 alpha	
36	Mm00434228_m1	Il1b	interleukin 1 beta	
37	Mm00446186_m1	Il1rn	interleukin 1 receptor antagonist	
38	Mm00434256_m1	Il2	interleukin 2	
39	Mm00517640_m1	Il21	interleukin 21	
40	Mm00600317_m1	Il21r	interleukin 21 receptor	
41	Mm01192969_m1	Il22ra2	interleukin 22 receptor, alpha 2	
42	Mm00519943_m1	Il23r	interleukin 23 receptor	
43	Mm00461162_m1	Il27	interleukin 27	
44	Mm00497259_m1	Il27ra	interleukin 27 receptor, alpha	
45	Mm00442885_m1	Il2rg	interleukin 2 receptor, gamma chain	
46	Mm00445259_m1	Il4	interleukin 4	
47	Mm00439646_m1	Il5	interleukin 5	
48	Mm00434284_m1	Il5ra	interleukin 5 receptor, alpha	

49	Mm00446190_m1	Il6	interleukin 6	
50	Mm00439653_m1	Il6ra	interleukin 6 receptor, alpha	
51	Mm00439665_m1	Il6st	interleukin 6 signal transducer	
52	Mm01290062_m1	Csf2	colony stimulating factor 2 (granulocyte-macrophage)	
53	Mm00655745_m1	Csf2rb	colony stimulating factor 2 receptor, beta, low-affinity (granulocyte-macrophage)	
54	Mm00469294_m1	Ebi3	Epstein-Barr virus induced gene 3	
55	Mm00475988_m1	Arg1	arginase, liver	
56	Mm00475162_m1	Foxp3	forkhead box P3	
57	Mm00440338_m1	Myd88	myeloid differentiation primary response gene 88	Enzymes, prostaglandins and adaptor proteins
58	Mm00440502_m1	Nos2	nitric oxide synthase 2, inducible	
59	Mm00479246_m1	Nox4	NADPH oxidase 4	
60	Mm00478374_m1	Ptgs2	prostaglandin-endoperoxide synthase 2	
61	Mm00439531_m1	Stat1	signal transducer and activator of transcription 1	
62	Mm01219775_m1	Stat3	signal transducer and activator of transcription 3	
63	Mm00448890_m1	Stat4	signal transducer and activator of transcription 4	
64	Mm01160477_m1	Stat6	signal transducer and activator of transcription 6	
65	Mm00477633_m1	Bcl6	B cell leukemia/lymphoma 6	
66	Mm00492590_m1	Ido1	indoleamine 2,3-dioxygenase 1	
67	Mm00500554_m1	Mmp12	matrix metallopeptidase 12	
68	Mm00485054_m1	Mmp14	matrix metallopeptidase 14 (membrane-inserted)	
69	Mm00476361_m1	Nfkb1	nuclear factor of kappa light polypeptide gene enhancer in B cells 1, p105	
70	Mm00479807_m1	Nfkb2	nuclear factor of kappa light polypeptide gene enhancer in B cells 2, p49/p100	
71	Mm00450960_m1	Tbx21	T-box 21	
72	Mm00441891_m1	Cd40	CD40 antigen	
73	Mm00441911_m1	Cd40lg	CD40 ligand	

74	Mm00711660_m1	Cd80	CD80 antigen	
75	Mm00444543_m1	Cd86	CD86 antigen	
76	Mm00486849_m1	Ctla4	cytotoxic T-lymphocyte-associated protein 4	
77	Mm03048248_m1	Cd274	CD274 antigen	
78	Mm00516023_m1	Icam1	intercellular adhesion molecule 1	
79	Mm00494862_m1	Icam2	intercellular adhesion molecule 2	Costimulatory and cell adhesion molecules
80	Mm00497600_m1	Icos	inducible T cell co-stimulator	
81	Mm00497237_m1	Icosl	icos ligand	
82	Mm00434513_m1	Itgb2	integrin beta 2	
83	Mm00486868_m1	Cd83	CD83 antigen	
84	Mm00493071_m1	Lag3	lymphocyte-activation gene 3	
85	Mm01285676_m1	Pdcd1	programmed cell death 1	
86	Mm00454540_m1	Havcr2	hepatitis A virus cellular receptor 2	
87	Mm00441242_m1	Ccl2	chemokine (C-C motif) ligand 2	
88	Mm00441258_m1	Ccl3	chemokine (C-C motif) ligand 3	
89	Mm00443111_m1	Ccl4	chemokine (C-C motif) ligand 4	
90	Mm01302427_m1	Ccl5	chemokine (C-C motif) ligand 5	
91	Mm00443113_m1	Ccl7	chemokine (C-C motif) ligand 7	
92	Mm01216147_m1	Ccr1	chemokine (C-C motif) receptor 1	
93	Mm01216173_m1	Ccr2	chemokine (C-C motif) receptor 2	
94	Mm01216171_m1	Ccr5	chemokine (C-C motif) receptor 5	Chemokines and chemokine receptors
95	Mm01301785_m1	Ccr7	chemokine (C-C motif) receptor 7	
96	Mm04207460_m1	Cxcl1	chemokine (C-X-C motif) ligand 1	
97	Mm00445235_m1	Cxcl10	chemokine (C-X-C motif) ligand 10	
98	Mm00436450_m1	Cxcl2	chemokine (C-X-C motif) ligand 2	
99	Mm00434946_m1	Cxcl9	chemokine (C-X-C motif) ligand 9	
100	Mm00438258_m1	Cxcr2	chemokine (C-X-C motif) receptor 2	
101	Mm00438259_m1	Cxcr3	chemokine (C-X-C motif) receptor 3	

102	Mm00434772_m1	Xcl1	chemokine (C motif) ligand 1	
103	Mm00441260_m1	Ccl9	chemokine (C-C motif) ligand 9	
104	Mm00444533_m1	Cxcl13	chemokine (C-X-C motif) ligand 13	
105	Mm00469712_m1	Cxcl16	chemokine (C-X-C motif) ligand 16	
106	Mm01701838_m1	Cxcl3	chemokine (C-X-C motif) ligand 3	
107	Mm00432086_m1	Cxcr5	chemokine (C-X-C motif) receptor 5	
108	Mm00442346_m1	Tlr2	toll-like receptor 2	Toll-like receptors
109	Mm01207404_m1	Tlr3	toll-like receptor 3	
110	Mm00445273_m1	Tlr4	toll-like receptor 4	
111	Mm00446590_m1	Tlr7	toll-like receptor 7	
112	Mm00446193_m1	Tlr9	toll-like receptor 9	

PANEL 2.

N°	Assay ID	GEN	NOMBRE DEL GEN	GRUPO
1	Mm00437762_m1	B2m	beta-2 microglobulin	Endogenous gene expression
2	Mm00446968_m1	Hprt	hypoxanthine guanine phosphoribosyl transferase	
3	Mm01201237_m1	Ubc	ubiquitin C	
4	Mm01331626_m1	Akt1	thymoma viral proto-oncogene 1	MAPK signaling pathway
5	Mm01173094_m1	Akt2	thymoma viral proto-oncogene 2	
6	Mm00442194_m1	Akt3	thymoma viral proto-oncogene 3	
7	Mm01973540_g1	Mapk3, Erk1	mitogen-activated protein kinase 3	
8	Mm00442479_m1	Mapk1, Erk2	mitogen-activated protein kinase 1	
9	Mm00489514_m1	Mapk8, Jnk	mitogen-activated protein kinase 8	
10	Mm00444968_m1	Mtor	mechanistic target of rapamycin (serine/threonine kinase)	
11	Mm01301009_m1	Mapk14, P38mapk	mitogen-activated protein kinase 14	
12	Mm01282781_m1	Pik3r1, Pi3k	phosphatidylinositol 3-kinase, regulatory subunit, polypeptide 1 (p85 alpha)	
13	Mm00440940_m1	Pparg	peroxisome proliferator activated receptor gamma	Lipid metabolism
14	Mm00443451_m1	Nr1h3	nuclear receptor subfamily 1, group H, member 3	
15	Mm00447040_m1	Pla2g4a	phospholipase A2, group IVA (cytosolic, calcium-dependent)	
16	Mm00447271_m1	Ptgis	prostaglandin I2 (prostacyclin) synthase	
17	Mm00441185_m1	Rxra	retinoid X receptor alpha	
18	Mm00436051_m1	Ptger2, Ep	prostaglandin E receptor 2 (subtype EP2)	Protaglandine synthesis
19	Mm00436053_m1	Ptger4	prostaglandin E receptor 4 (subtype EP4)	
20	Mm00452105_m1	Ptges	prostaglandin E synthase	
21	Mm00460181_m1	Ptges2	prostaglandin E synthase 2(Ptges2)	
22	Mm01731378_g1	Ptges3	prostaglandin E synthase 3 (cytosolic)	
23	Mm00477214_m1	Ptgs1	prostaglandin-endoperoxide synthase 1	

24	Mm01199500_m1	P2rx7	purinergic receptor P2X, ligand-gated ion channel, 7	
25	Mm00435472_m1	P2ry2	purinergic receptor P2Y, G-protein coupled 2	
26	Mm00436055_m1	Ptgfr	prostaglandin F receptor	
27	Mm00479846_m1	Hpgds, Ptgds2	intercellular adhesion molecule 1	
28	Mm00482476_m1	Ptgr1, Ltb4dh	prostaglandin reductase 1	
29	Mm00521839_m1	Ltb4r1, Blt1	leukotriene B4 receptor 1	
30	Mm01182747_m1	Alox5	arachidonate 5-lipoxygenase	
31	Mm00507789_m1	Alox15	arachidonate 15-lipoxygenase	
32	Mm00521826_m1	Lta4h	leukotriene A4 hydrolase	
33	Mm00545833_m1	Alox12	arachidonate 12-lipoxygenase	
34	Mm00839636_g1	Cd68	CD68 antigen	Cholesterol pathway
35	Mm00432403_m1	Cd36	CD36 antigen	
36	Mm00459972_m1	Cd209d	CD209d antigen	C-Type Lectin Receptors (CLRs)
37	Mm01183349_m1	Clec7a	C-type lectin domain family 7, member a	
38	Mm01329362_m1	Mrc1	mannose receptor, C type 1	
39	Mm01183378_m1	Cd69	CD69 antigen	
40	Mm00495182_m1	Klrd1, Cd94	killer cell lectin-like receptor, subfamily D, member 1	
41	Mm00435587_m1	Pfkl	phosphofructokinase, liver, B-type	Carbohidrates synthesis
42	Mm00439344_m1	Hk1	hexokinase 2	
43	Mm00443385_m1	Hk2	hematopoietic prostaglandin D synthase	
44	Mm00441480_m1	Slc2a1	solute carrier family 2 (facilitated glucose transporter), member 1	
45	Mm00472712_m1	Gys1	granzyme B	
46	Mm00504650_m1	Pfkfb3	6-phosphofructo-2-kinase/fructose-2,6-biphosphatase 3	
47	Mm00554300_m1	Pdk1	pyruvate dehydrogenase kinase, isoenzyme 1	
48	Mm01268229_m1	Pgm2	phosphoglucomutase 2	
49	Mm01612132_g1	Ldha	lactate dehydrogenase A	
50	Mm00833691_g1	Tpi1	triosephosphate isomerase 1	
51	Mm00434151_m1	Il10ra	interleukin 10 receptor, beta	

52	Mm00446726_m1	Il13ra1	interleukin 13 receptor, alpha 2	Interleukines and interleukin receptors
53	Mm00515166_m1	Il13ra2	interleukin 15	
54	Mm00434210_m1	Il15	interleukin 16	
55	Mm00516039_m1	Il16	interleukin 17 receptor A	
56	Mm00515178_m1	Il18r1	interleukin 18 receptor accessory protein	
57	Mm00516053_m1	Il18rap	interleukin 1 alpha	
58	Mm00663697_m1	Il22ra1	interleukin 23, alpha subunit p19	
59	Mm01340213_m1	Il27ra	interleukin 2 receptor, alpha chain	
60	Mm00434268_m1	Il2rb	interleukin 2 receptor, gamma chain	
61	Mm01275139_m1	Il4ra	interleukin 5 receptor, alpha	
62	Mm00434295_m1	Il7r, Cd127	interleukin 9 receptor	
63	Mm00434313_m1	Il9r	interferon regulatory factor 1	
64	Mm00499822_m1	Il25	interleukin 25	
65	Mm00434305_m1	Il9	interleukin 9	
66	Mm00499822_m1	Il2ra, Cd25	interleukin 2 receptor, alpha chain	
67	Mm00444241_m1	Il22	interleukin 22	
68	Mm00516136_m1	Ccl17	chemokine (C-C motif) ligand 17	Chemokines and chemokine receptors
69	Mm00839966_g1	Ccl19	chemokine (C-C motif) ligand 19	
70	Mm00436439_m1	Ccl22	chemokine (C-C motif) ligand 22	
71	Mm00438271_m1	Ccr4	chemokine (C-C motif) receptor 4	
72	Mm00444662_m1	Cxcl11	chemokine (C-X-C motif) ligand 11	
73	Mm01292123_m1	Cxcr4	chemokine (C-X-C motif) receptor 4	
75	Mm00472858_m1	Cxcr6	Epstein-Barr virus induced gene 3	
76	Mm00441263_m1	Cxcl15	chemokine (C-X-C motif) ligand 15	
77	Mm00490880_m1	Stat2	signal transducer and activator of transcription 2	Enzymes and adaptor proteins
78	Mm00600614_m1	Jak1	Janus kinase 1	
79	Mm01208489_m1	Jak2	Janus kinase 2	
80	Mm00477631_m1	Bcl2	B cell leukemia/lymphoma 2	

81	Mm00432050_m1	Bax	BCL2-associated X protein	
82	Mm00437783_m1	Bcl2l1	BCL2-like 1	
83	Mm00438861_m1	Fadd	Fas (TNF receptor superfamily member 6)	
84	Mm00442834_m1	Gzmb	hepatitis A virus cellular receptor 2	
85	Mm00436979_m1	Tgm2	transglutaminase 2, C polypeptide	
86	Mm00445109_m1	Retnla, Fizz1	resistin like alpha	
87	Mm00448427_m1	Ptpn1, Ptp1b	protein tyrosine phosphatase, non-receptor type 1	
88	Mm01278617_m1	Mki67, Ki67	antigen identified by monoclonal antibody Ki 67	
89	Mm00661498_m1	CD57, B3gat1	beta-1,3-glucuronyltransferase 1 (glucuronosyltransferase P)	
90	Mm00451734_m1	Pdcd1lg2	programmed cell death 1 ligand 2	
91	Mm00599683_m1	Cd3e	CD3 antigen, epsilon polypeptide	
92	Mm01182108_m1	Cd8a	CD8 antigen, alpha chain	
93	Mm00488332_m1	Siglec1, Cd169	sialic acid binding Ig-like lectin 1, sialoadhesin	
94	Mm00434455_m1	Itgam	integrin alpha M	Costimulatory and cell adhesion molecules
95	Mm00483137_m1	Cd28	CD28 antigen	
96	Mm00801807_m1	Itgal	integrin alpha M	
97	Mm00444461_m1	Cd160	CD160 antigen	
98	Mm01251919_m1	Itgae, Cd103	integrin alpha L	
99	Mm00442754_m1	Cd4	CD4 antigen(Cd4)	
100	Mm01149710_m1	Ncam1, Cd56	neural cell adhesion molecule 1	
101	Mm00484683_m1	Gata3	glycogen synthase 1, muscle	
102	Mm00467257_m1	Nfat5	nuclear factor of activated T cells 5	Transcription factors
103	Mm00468869_m1	Hif1a	hexokinase 1	
104	Mm00627599_m1	Ucp2	uncoupling protein 2 (mitochondrial, proton carrier)	Carrier proteins
105	Mm00437136_m1	Tnfrsf18	tumor necrosis factor receptor superfamily, member 18	
106	Mm00616981_m1	Btla	B and T lymphocyte associated	Cytokines and cytokine receptors
107	Mm00438864_m1	Fasl	forkhead box P3	
108	Mm01204974_m1	Fas	Fas ligand (TNF superfamily, member 6)	

109	Mm00442039_m1	Tnfrsf4	tumor necrosis factor receptor superfamily, member 4	
110	Mm00440228_gH	Lta	lymphotoxin A	
111	Mm01342740_g1	Socs1	suppressor of cytokine signaling 1	
112	Mm01249143_g1	Socs3	suppressor of cytokine signaling 3	

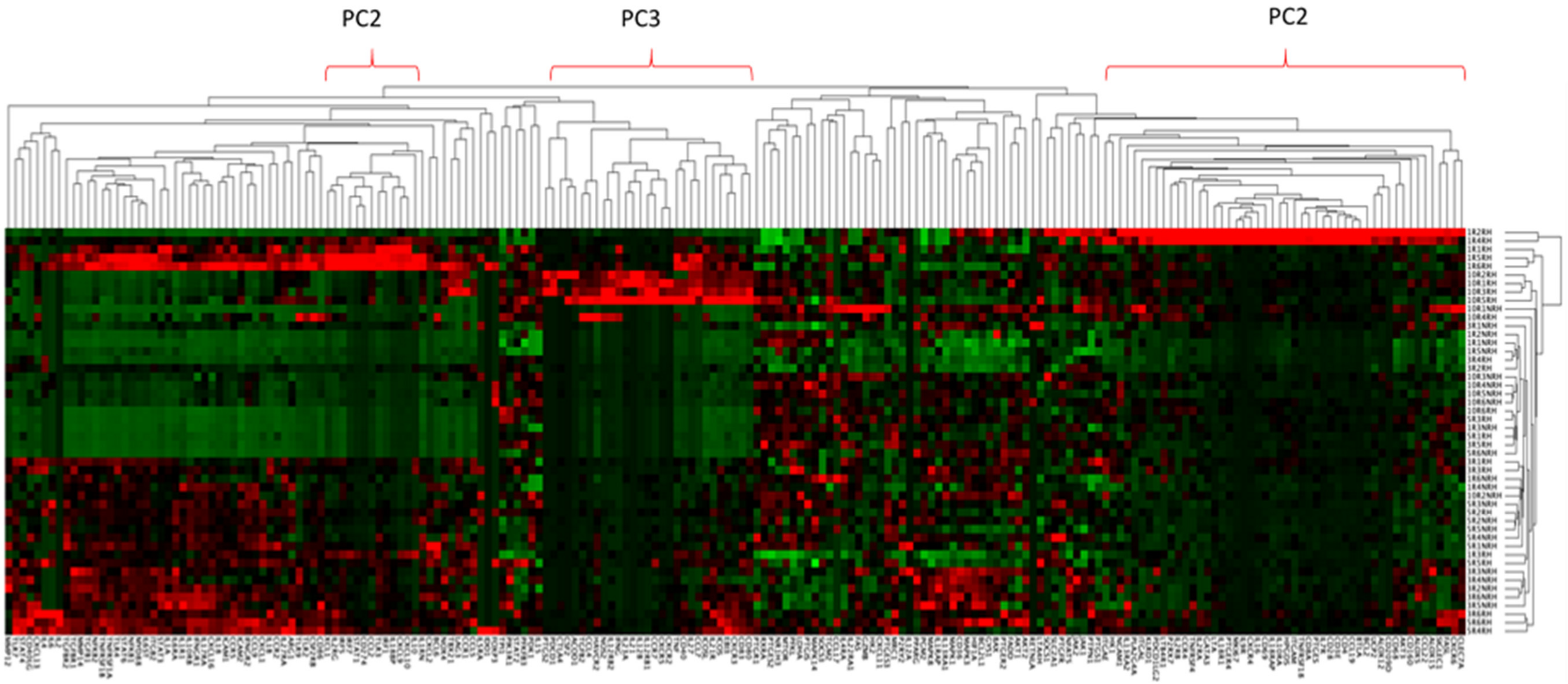

Figure S1. Unsupervised hierarchal clustering of gene expression data. qPCR was carried out on mice liver tissue infected and control with *L. infantum* at 1, 3, 5 and 10 dpi. Unsupervised hierarchal clustering of the complete NRQ data: samples vs genes, was applied and based on Euclidean distance and pairwise average-complete linkage. Genes downregulated during infection are shown in green and upregulated genes are in red.
